# Towards an immunoscore for triple negative breast cancer (TNBC): lymphocytic infiltrate predicts outcome

**DOI:** 10.1186/2051-1426-1-S1-O22

**Published:** 2013-11-07

**Authors:** Sylvia Adams, Robert Gray, Sandra Demaria, Lori J Goldstein, Edith A Perez, Lawrence N Shulman, Silvana Martino, Nancy E Davidson, George W Sledge, Joseph A Sparano, Sunil Badve

**Affiliations:** 1 Eastern Cooperative Oncology Group, Philadelphia, PA, USA; 2 North Central Cancer Treatment Group, Rochester, MN, USA; 3 Cancer and Leukemia Group B, Chicago, IL, USA; 4 Southwest Oncology Group, Portland, OR, USA

## Background

Efforts are ongoing to further classify solid tumors by immunoscores, which have prognostic implications and may also have predictive value and identify targets for novel therapies to increase cure rates. TNBC are highly aggressive breast cancers, which are frequently infiltrated by lymphocytes, recently linked to prognosis in lymph node (LN)-positive TNBC.

## Methods

Full face hematoxylin & eosin (H&E)-stained tumor sections of 500 randomly selected TNBC (LN positive and negative) from two adjuvant US clinical trials (E1199, E2197) were assessed for density and location of TILs. Associations with outcomes after adjuvant chemotherapy were determined and models were performed to identify relationships with established prognostic factors.

## Results

481 tumors were evaluable, stromal tumor infiltrating lymphocytes (sTIL) were observed in 80% of cancers; 49%, 27% and 4% of tumors had a 10%, 20-40% and 50% or greater infiltrate, respectively. With a median follow-up of 10.6 years the degree of sTIL was positively correlated with disease-free survival (DFS), distant recurrence-free interval and overall survival. There were associations between sTIL scores and LN status but not age, menopausal status or tumor size. The likelihood of a sTIL score of 0 decreased as the number of positive LN increased, with a rate of 25.4% (50/197) for LN negative breast cancers, 18.1% (36/50) for tumors with 1-3 positive LN and 10.6% (9/85) for tumors with >3 LN (P=0.01). For DFS, the estimated HR was 0.84 (0.74, 0.95, P=0.005) for a 10 point change in sTIL, which increased further in significance when LN involvement (0 vs 1-3 vs >3) was taken into account (HR 0.82; 0.72,0.94, P=0.003).

## Conclusion

We demonstrated that sTIL can improve outcome prediction in TNBC beyond traditional TNM staging. Our data provide clinical validation of recent findings by Loi et al, JCO 2013 using identical methods. We therefore propose that evaluation of stromal lymphocytic infiltrates in H&E-stained tumor sections provides a simple method to establish an immunoscore for this subtype of breast cancer.

